# Characteristics, Relationships, and Differences in Muscle Activity and Impact Load Attenuation During Tennis Forehand Stroke with Different Grips

**DOI:** 10.3390/life14111433

**Published:** 2024-11-06

**Authors:** Rui Dong, Xinyu Su, Shichen Li, Xindi Ni, Ye Liu

**Affiliations:** School of Sport Science, Beijing Sport University, Beijing 100084, China; dongrui0223@163.com (R.D.); xinyu_su0405@163.com (X.S.); lishichen0705@163.com (S.L.); nxd_bsu@foxmail.com (X.N.)

**Keywords:** tennis, forehand stroke, different grips, muscle activity, impact load, cross-correlation

## Abstract

In forehand strokes with different grips in tennis, the forearm muscle activities, the distribution and attenuation of the impact loads, and the effects of the muscles on the impact load attenuation exhibited different characteristics. This study aimed to explore these characteristics by analyzing electromyography (EMG) and acceleration data, and comparing the differences between the Eastern and Western grips. Fourteen level II or above tennis players (ten males, aged 22.4 ± 3.6 years; four females, aged 19.8 ± 2.0 years) were recruited and instructed to perform forehand strokes using the Eastern and Western grips, respectively. The EMG of eight forearm muscles and the acceleration data at the ulnar and radial sides of the wrist and elbow were collected. The root mean square (RMS), the peaks of the impact load, the amplitude of impact load attenuation (AC), and the jerk value (Jerk) were calculated. The cross-correlation coefficients and time delays of EMG–EMG, EMG–AC, and EMG–jerk were obtained using the cross-correlation method. The results showed that in the Eastern grip group (group E), the RMS of the flexor carpi ulnaris (FCU) was significantly greater than that in the Western grip group (group W). In group E, the peaks of impact load, AC, and Jerk on the Y axis of the wrist ulnar side were all significantly higher than those in group W. The activity of the extensor digitorum commonis (EDC) had significantly different effects on the amplitude and rate of impact load attenuation at specific locations in different grips, especially at the elbow (*p* < 0.05). The conclusion indicated that the FCU exhibited higher levels of EMG activity in the Eastern grip. This grip responded to greater impact loads with more substantial and rapid attenuation on the wrist ulnar side. Furthermore, the EDC appeared to contribute more to the amplitude of impact load attenuation in the Western grip and to have a more significant influence on the rate of impact load attenuation in the Eastern grip, especially at the elbow. These results suggest that tennis players and coaches should pay more attention to improving the strength of the EDC and FCU, which can improve sports performance and comfort, as well as prevent sports injuries.

## 1. Introduction

Tennis is the most popular racket sport played worldwide [1] in 215 countries [2]. The number of people participating in tennis, both recreationally and competitively, has grown significantly over the past two decades [1]. In a match, a tennis player may hit over 1100 groundstrokes [3], and the vibrations caused by the strokes are transmitted directly to the body. This constant transmission of vibrations can be a critical factor affecting sports performance and comfort, and may even cause injury [4].

There are three main stroke skills in tennis. They are called serve, forehand stroke, and backhand stroke [5]. The forehand stroke is the most frequently used groundstroke skill in professional tennis [6] and is usually the first stroke learned by amateur tennis players [7]. The forehand stroke consists of four different grips: continental, Eastern, semi-Western, and full Western. The continental grip is only used in serves and volleys at present [8]. As for the other three grips, the number of people using them varies for different reasons [1]. In competition, proper stroke technique is essential for performance to achieve favorable outcomes [9] (e.g., scoring points or forced errors) and reduce the risk of overuse and acute injuries. However, different grips have unique characteristics and stroke performance [9,10]. The Eastern grip forehand stroke is characterized by a higher percentage of impact force directed forward, which means that the ball speed is comparatively high and the spin is relatively low [9,10]. In the Western grip, the weight of the force is relatively high vertically. Therefore, the spin is higher, while the ball speed is relatively low [9,10]. The semi-Western grip combines the characteristics of the Eastern and Western grips, so the ball speed and spin are moderate. Meanwhile, a study of 370 tennis players with wrist injuries [1] showed that both the semi-Western and Western grips showed similar results (e.g., more ulnar injuries). In comparison, the Eastern grip showed more radial injuries. Therefore, the Eastern and Western grips are more representative regarding technical characteristics and injury patterns.

Currently, research on tennis focuses on several significant aspects, such as kinematics [11,12,13,14], electromyographic activity [14,15,16], impact loading [17,18], and teaching [9]. More EMG-related research is focused on the correspondence between sports injuries and EMG signal characteristics [19,20]. The EMG research on tennis skills analysis is not yet detailed, and most studies are holistic, including several body segments. Rota et al. investigated the relationship between muscular coordination and ball speed in forehand groundstrokes [16]. They selected ten muscles from the trunk to the upper limb but only included two forearm muscles: the flexor carpi radialis (FCR) and the extensor carpi radialis (ECR). Similarly, in the research by Tai et al. [21] and Furuya et al. [22] on the volley and other stroke skills, they also only selected the FCR and ECR for study. Even though Loushin et al. [23] later established a biomechanical database for forehand and two-handed backhand strokes of adolescent tennis players, which included kinematics and EMG data from the elbow, wrist, and forearm. However, for the forearm, only the ECR, FCR, extensor carpi ulnaris (ECU), and flexor carpi ulnaris (FCU) were collected for EMG signals, and no more detailed research was conducted on other muscles. In addition, although there have been many EMG studies of forehand strokes, fewer of these studies have focused on different grips. Abuwarda et al. [15] investigated the muscle activations of the dominant arm during forehand stroke of wheelchair tennis players and compared the differences between the various phases of the movement. Among the target muscles involved in this study, only two forearm muscles (FCR and ECR) were included. The results showed that the FCR and ECR were well synchronized during all phases of forehand stroke. However, the level of muscle activity varies at different phases. Although Yeh. et al. [24] performed an EMG test of the forehand stroke of healthy tennis players, this study also only included two forearm muscles. Obviously, selecting only two muscles in the forearm for research will not yield the desired results. To address the shortcomings of previous studies, we selected eight forearm muscles for analysis and compared the differences in muscle activity during forehand strokes with the Eastern and Western grips.

The impact between the ball and the racket will generate powerful shock and vibration waves. It can lead to increased strain on the tendons around the elbow joint [25], which can reduce sports performance and cause injuries to the limb. After hitting the ball, many factors affect the transmission of shock and vibration waves, such as tennis speed, racket head velocity, stiffness of the racket string, impact locations, and grip force [26,27,28,29]. Rogowski et al. [18] investigated the effects of two different tennis rackets (low and high vibration rackets) and forehand stroke velocities on racket vibration and its transmission to the wrist joint of the athlete. It was found that with increasing stroke velocity, the amount of racket and wrist joint vibration increased, but the frequency-related content was altered weakly. Yeh et al. [24] tested forearm muscle activity and the vibration wave transmission from racket to forearm during forehand strokes, using rackets with and without a novel vibration damping technology (VDT). However, studies on the characteristics of impact load transmission in different grip styles are still relatively rare.

In addition, soft tissues [30] and muscle activation [31] effectively reduce impact loads, which can largely avoid injury to the human body caused by impact loads. In a study of arm vibration induced by backhand strokes, Hennig et al. [17] found that the amplitude and integral of acceleration decreased more than fourfold from the wrist to the elbow joint. Moreover, during backhand strokes, elite athletes had less forearm vibration and lower EMG amplitude of the wrist extensor muscles compared to novices, suggesting that the occurrence of injuries in tennis may also be related to the inappropriate use of technical skills. In the Eastern grip, the forearm is in a pronation position with ulnar deviation of the wrist joint. In contrast, the Western grip shows supination of the forearm and radial deviation of the wrist joint. These morphological and structural differences may lead to different forearm muscle activation patterns and shockwave resistance mechanisms between the Eastern and Western grips [32]. In an anatomical study conducted by Tagliafico et al. [8] on non-elite junior tennis players with high-resolution ultrasound, no association was found between shoulder and biceps injuries and different grips. However, after 12 weeks of centrifugal training of forearm muscles in a tennis player with severe ulnar-sided pain, Knobloch et al. found that the patient’s forearm pain virtually disappeared [33]. Therefore, it is reasonable to believe that forearm muscles play a vital role in tennis, both for the performance of stroke and the transmission of impact loads [34,35]. It is essential to analyze and study the activity pattern of forearm muscles, the change of impact load, and their relationship in forehand tennis strokes with different grips.

In this study, we aim to explore the activation of forearm muscles, the changes in impact loads, and their correlation during tennis forehand strokes with Eastern and Western grips. These findings can more deeply explain the differences in the forearm muscle contraction patterns, the impact load distribution, and the impact resistance in arms with different grips. Furthermore, our study provides a theoretical basis for tennis coaches and athletes to target training program design, improve sports performance, and prevent sports injuries. We hypothesized that the forearm muscle activations are different in forehand strokes with two different grips. In the Eastern grip, the wrist flexor muscles may be more activated, while in the Western grip, the wrist extensor muscles may be more activated. Furthermore, the distribution and attenuation characteristics of impact loads may also differ. In the Eastern grip, the wrist radial side may suffer greater impact loads; in the Western grip, the impact loads may be distributed more on the wrist ulnar side. At the same time, there are also several differences between the two groups in the correlation between muscle activity and attenuation of impact loads.

## 2. Materials and Methods

### 2.1. Subjects

The PASS 15.0 software was used to estimate the sample size of the subjects (α = 0.05, power = 0.8), and the result showed that the minimum sample size was 14. Since high-level athletes are scarce, we tried our best to recruit 15 subjects to participate, and 14 subjects finished the tests. All of them were tennis players at Beijing Sport University (ten males, aged 22.4 ± 3.6 years, height 179.9 ± 6.9 cm, weight 78.5 ± 5.9 kg, training period 11.3 ± 3.1 years; four females, aged 19.8 ± 2.0 years, height 169.9 ± 3.2 cm, weight 56.4 ± 5.9 kg, and training period 11.3 ± 3.1 years). The dominant hand of all subjects was their right. The inclusion criteria for the subjects were as follows: (1) National tennis player of level II or above (the possession of a sports classification certificate issued by the authoritative organization is the criterion for determining the subject’s level, which means the athlete has previously achieved a ranking in official competition); (2) Adults aged 18 to 26 years old, regardless of gender; (3) The subject has not experienced any sports injuries or surgery in the past 6 months. (4) All subjects had not participated in any training or competition and did not feel fatigued or discomfort within 48 h before the experiment. Furthermore, there was no record of any medication use. Any subject who did not meet the inclusion criteria was excluded. The Ethics Committee of Beijing Sport University approved our study. All subjects participated in the experiment voluntarily and signed an informed consent form before the experiment.

### 2.2. Experimental Protocol

All of the tests in this study were conducted at the No. 3 court of Beijing Sports University Tennis Comprehensive Training Center. All subjects used the same racket and several tennis balls of the same brand and type for the test (BABOLAT Pure Drive tennis racket, TAAN TT8600 tennis strings, ODEA Silver tennis balls, 52 lbs. vertical stringing, 54 lbs. horizontal stringing).

#### 2.2.1. Warm-Up and Maximum Drive Velocity Test

First, our researcher provided subjects with a detailed introduction to the technical characteristics of the Eastern and Western grips (Supplementary Figure S1) and the specific procedures of the experiment. All subjects participated in a standardized warm-up, including forehand stroke practice using the Eastern and Western grips. Each grip was practiced in at least three groups for 10 shots. After a sufficient warm-up, the maximum drive velocity was tested. After the automatic pitching machine shot the ball out, subjects hit the ball to the target area (Supplementary Figure S2) at full power 10 times with their usual grip. The five strokes with the highest ball speed were selected, and their average value was calculated as the subject’s maximum drive velocity. The Stalker PRO II radar speed detector (Stalker Pro II, Stalker Radar, Plano, TX, USA) was used to measure the ball speed on each stroke, with the mode selected as tennis. After the subjects had fully rested, the wireless surface EMG sensors and accelerometers were placed.

#### 2.2.2. Placement of Wireless EMG System and Accelerometer

Surface EMG is widely used in assessing training, sports medicine, and other areas, and it also shows great potential in many novel regions [36]. In this research, the Delsys wireless EMG system (DelsysTrigno, Boston, MA, USA) was used to record forearm muscle activity during the forehand stroke action, with a sampling frequency of 2000 Hz. Before the experiment began, our professional researcher used ultrasonic image recognition technology to locate the surface positions of eight muscles for each subject. These eight muscles are the brachioradialis (BR), flexor carpi radialis (FCR), flexor digitorum superficialis (FDS), pronator teres (PT), flexor carpi ulnaris (FCU), extensor digitorum communis (EDC), extensor carpi radialis (ECR), and extensor carpi ulnaris (ECU). Skin preparation included shaving and wiping the skin with alcohol. Electrode placements followed the recommendations of the ISEK tutorials (Merletti and Cerone, 2020) [36]. The electrode is parallel to the long axis of the muscle fiber and placed at the most prominent point of the muscle belly. The surface EMG sensors were bound with a medical elastic band to minimize the occurrence of motion artifacts.

Accelerometers are commonly used to estimate impact loading [37]. We used three triaxial wired accelerometers (YIYANG, YSV8016, Beijing, China) to collect acceleration signals from the wrist and elbow joint, with a sampling frequency of 1024 Hz. Acceleration sensors were placed on the skin surface of the subject’s leading arm at the styloid process of the ulna, the malleolus radialis, and the external epicondyle of the humerus. The X-axis was parallel to the long axis of the forearm, the Y-axis was parallel to the coronal axis (representing the medial–lateral directions), and the Z-axis was parallel to the sagittal axis (representing the anterior–posterior directions). The accelerometers were bound using a medical elastic bandage to reduce the effect of soft tissue vibration. The same professional researcher performed all of the above tasks.

#### 2.2.3. Forehand Stroke Test with Different Grips

The subject stood in a prescribed place and hit the ball with full force along a prescribed route according to a particular grip sequence. The grip sequence was obtained by random sampling. The drive velocity was measured using a radar speed detector. A stroke was considered valid if the drive velocity reached more than 85% of the subject’s maximum drive velocity and the ball landed in the target area as defined (Supplementary Figure S2). When three valid data points were obtained for each grip, the test was terminated. Subjects were verbally motivated and corrected for errors during the test to achieve optimal performance.

### 2.3. Data Processing

EMG and acceleration signals were analyzed using a self-written program in MATLAB R2023a (The MathWorks Inc., Natick, MA, USA). First, the EMG and acceleration signals were demeaned. The EMG signal was filtered using a 4th-order Butterworth band-pass filter (20–500 Hz) to reduce motion artifacts and high-frequency noise, while the acceleration signal was filtered (15–400 Hz) [28,34] to eliminate the very high-frequency vibrations of the racket frame and the low-frequency components owing to arm movement after the impact. Then, we selected 200 ms (containing all information on impact attenuation) [34] after the moment of stroke for analysis to obtain indicators including the root mean square (RMS), peak acceleration (PEAK–acc), peak acceleration change (PAC–acc), and peak jerk (PEAK–jerk).

After that, we performed cross-correlation analysis on the EMG and acceleration signals and obtained the cross-correlation coefficients of the different forearm muscles (rEMG–EMG), the EMG and acceleration change (rEMG–AC), the EMG and Jerk (rEMG–jerk), as well as their time delays (ΔtEMG–EMG, ΔtEMG–AC, ΔtEMG–jerk).

#### 2.3.1. EMG

In our study, the EMG activity of the muscles was quantified using the root mean square value. The root mean square (RMS) is defined as:

(1) $$RMS=\sqrt{\frac{1}{N}\sum_{i=1}^{N}x_{i}^{2}}$$

#### 2.3.2. Acceleration

The peak acceleration (PEAK–acc) is a standard time–domain index for impact loads, representing the maximum impact load received by the human body in sports.

During the intercepted time, we took the absolute value of the difference between the acceleration and peak acceleration values at each time point as the amplitude of acceleration change (AC_acc_). In our study, it can represent the amplitude of the acceleration change at that moment. The AC_acc_ is defined as follows:

(2) $${AC}_{acc}=\left|T_{acc}-P_{acc}\right|$$

Here, T_acc_ is the acceleration value at a moment on an axis, and P_acc_ is the peak acceleration for this axis.

We calculated the derivative of the acceleration signal and took its absolute value to obtain the rate of change of acceleration at a certain moment, called Jerk. Jerk is often used to evaluate an action’s smoothness [38] and can represent the rate or smoothness of impact attenuation in our study. The peak jerk (PEAK–jerk) is the maximum jerk value during the intercepted time. Jerk is defined as follows:

(3) $$Jerk=\left|\frac{dAcc}{dt}\right|$$

Here, Acc represents the acceleration signal, and t is time.

#### 2.3.3. Cross-Correlation

Cross-correlation is a relatively mature method for analyzing the degree of correlation between two time–domain signals under a certain time delay. In the processing of surface EMG signals, cross-correlation is an effective tool for quantifying the coordinated work between two muscles [39]. The cross-correlation still performs well even when studying two different time–domain signals [40,41]. The cross-correlation coefficient and time delay are two main indicators of cross-correlation analysis. An increasing coefficient and decreasing time delay mean two time–domain signals are more synchronized [42]. The cross-correlation coefficient was interpreted as follows: excellent (0.80–1.00), strong (0.60–0.79), moderate (0.40–0.59), weak (0.20–0.39), and very weak (0.00–0.19) [43]. In this study, for the time delay of two time–domain signals, A and B (ΔtA–B), a positive value indicated that A lagged behind B, while a negative value meant that A was earlier than B.

In order to explore the coordinated work of different muscles and the relationship between muscle activity and impact load changes, we calculated the rEMG–EMG between all eight target muscles, the rEM–AC and rEMG–jerk between the EMG signals of the eight target muscles and the acceleration signals of the three triaxial acceleration sensors (nine sets of acceleration data), as well as their Δt. The cross-correlation coefficient and delay were obtained using the cross-correlation function. The cross-correlation of two time–domain signals (f and g) is defined as:

(4) $$\left(f*g\right)\left[n\right]=\sum_{-\infty }^{+\infty }f\left[m\right]*g[n+m]$$

Here, f and g are two time–domain signals containing m samples, and n represents the time delay.

### 2.4. Statistical Analysis

All statistical analyses were performed using IBM SPSS Statistics 26 (SPSS Inc., Chicago, IL, USA) in our study. The data were divided into two groups: Eastern grip and Western grip. First, the Shapiro–Wilk test was used to check the normality of all indicators. The Shapiro–Wilk test and histogram showed that only the RMS of all indicators presented a normal distribution; the other indicators did not conform to the normal distribution. Therefore, the independent-sample *t*-test was used to analyze RMS data that conformed to the normal distribution. The Mann–Whitney U test was used for other data that did not show a normal distribution. The significance level was set at *p* < 0.05. When reporting statistical results, we used the mean ± standard deviation to present the data that conformed to the normal distribution; data that did not conform to the normal distribution were presented as the median and interquartile.

## 3. Results

### 3.1. Root Mean Square (RMS)

**Figure 1:**
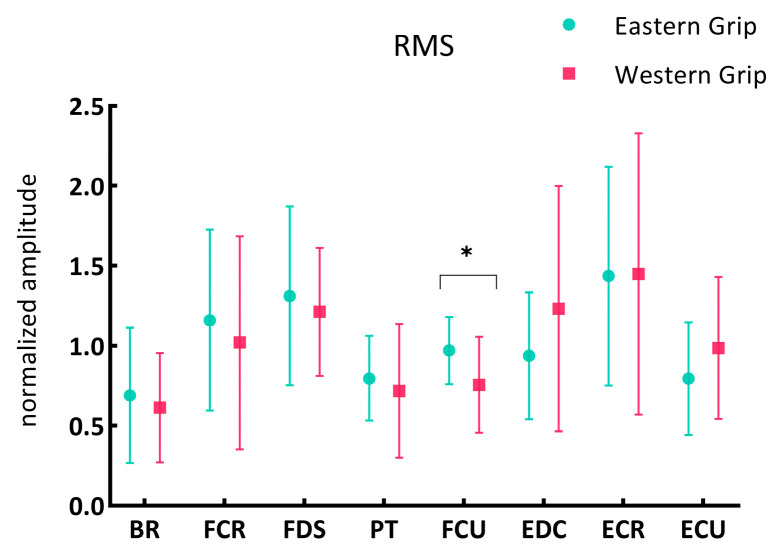
The RMS of the eight forearm muscles during forehand stroke with Eastern and Western grip. Data are presented as mean ± standard deviation. The * indicates a significant difference (*p* < 0.05). (BR: brachioradialis; FCR: flexor carpi radialis; FDS: flexor digitorum superficialis; PT: pronator teres; FCU: flexor carpi ulnaris; EDC: extensor digitorum communis; ECR: extensor carpi radialis; ECU: extensor carpi ulnaris; applies to all figures).

The results of the independent-sample *t*-test showed that (Figure 1 and all the results with significant differences summarized in Supplementary Table S1) there was a significant difference in the RMS of the FCU between the Eastern grip group (group E) and the Western grip group (group W) (*p* = 0.037). In group E, the RMS of FCU (0.97 ± 0.21) was higher than that of the group W (0.76 ± 0.30).

### 3.2. The Acceleration Indicators

#### 3.2.1. Peak Acceleration (PEAK–acc)

**Figure 2:**
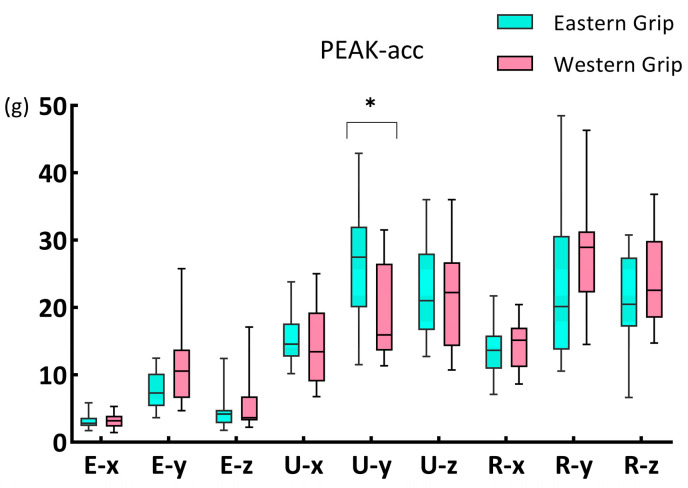
The PEAK–acc of the X, Y, and Z axes of three acceleration sensors during forehand stroke with Eastern and Western grip. The data are presented as a box plot with the median, interquartile range, maximum, and minimum values. The * indicates a significant difference (*p* < 0.05). E–x, U–x, and R–x represent the X-axis acceleration data of three acceleration sensors placed on the external epicondyle of the humerus, the styloid process of the ulna, and the malleolus radialis. E–y, U–y, and R–y represent the acceleration data of the Y-axis of three acceleration sensors. E–z, U–z, and R–z represent the Z-axis acceleration data of three acceleration sensors. Same below.

The results of the Mann–Whitney U test showed a significant difference in PEAK–acc of the Y-axis on the ulnar side (*p* = 0.016) between group E and group W (Figure 2). Group E (27.46, 11.99 [the median, the interquartile range; applies throughout]) had a higher PEAK–acc than group W (15.95, 12.93).

#### 3.2.2. Peak Amplitude of Acceleration Change (PAC–acc)

**Figure 3:**
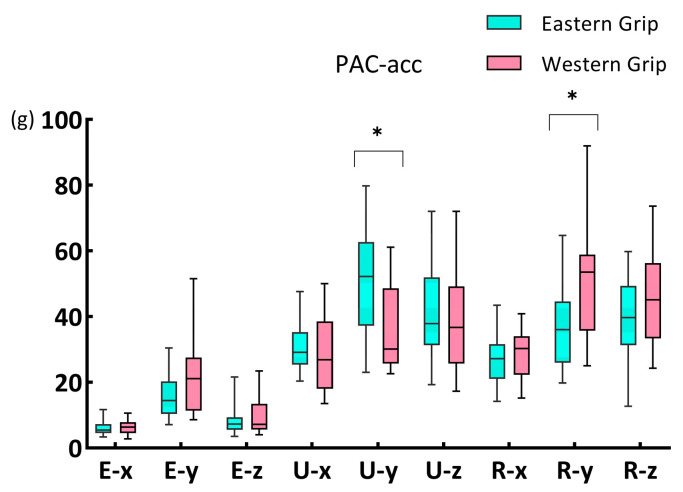
The PAC–acc of the X, Y, and Z axes of three acceleration sensors during forehand stroke with Eastern and Western grip. The data are presented as a box plot, and the * indicates a significant difference (*p* < 0.05).

The Mann–Whitney U test showed (Figure 3) significant differences between groups E and W in the PAC–acc of U–y (*p* = 0.008) and R–y (*p* = 0.048). For PAC–acc on U–y, group E (52.18, 25.55) was greater than group W (30.09, 22.96); on R–y, the PAC–acc of group W (53.58, 23.20) was greater than that of group E (36.03, 18.69).

#### 3.2.3. PEAK–jerk

**Figure 4:**
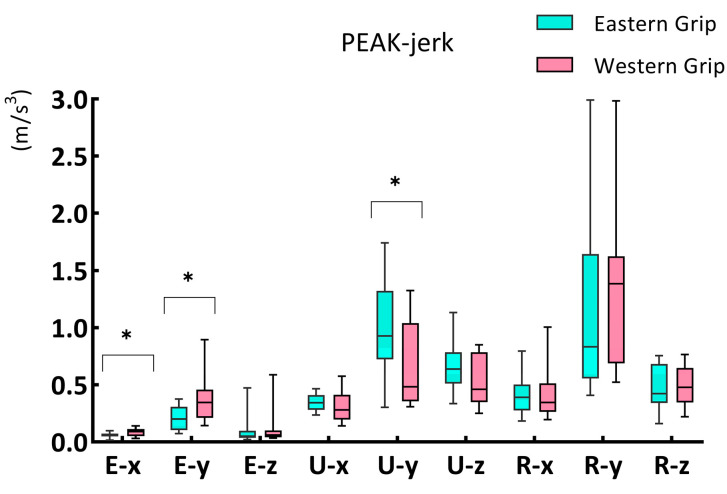
The PEAK–jerk of the X, Y, and Z axes of three acceleration sensors during forehand stroke with Eastern grip and Western grip. The data are presented as a box plot, and the * indicates a significant difference (*p* < 0.05).

The results showed that (Figure 4) there were significant differences in PEAK–jerk between groups E and W, including E–x (*p* = 0.048), E–y (*p* = 0.024), and U–y (*p* = 0.021). At E–x, the PEAK–jerk of group W (0.090, 0.062) was higher than that of group E (0.057, 0.024). Similarly, at E–y, the value of group W (0.346, 0.247) was also higher than that of group E (0.202, 0.205). However, the results were opposite in U–y, where the PEAK–jerk of group W (0.485, 0.683) was much lower than that of group E (0.929, 0.599).

### 3.3. The Cross-Correlation Coefficients and Time Delays of EMG and Acceleration Change

#### 3.3.1. General Description

**Figure 5:**
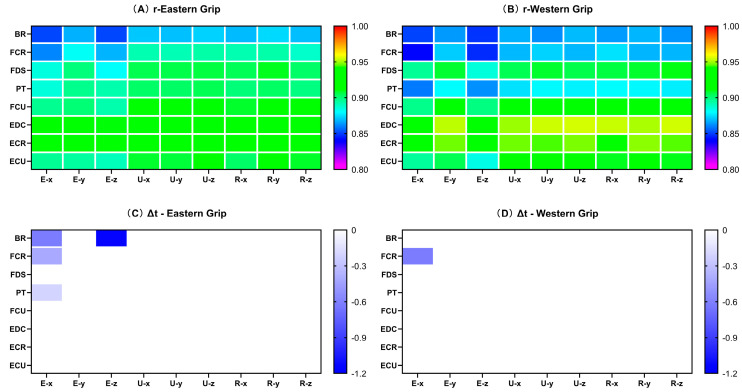
The cross-correlation coefficients and delays of EMG–AC. Each small square represents the correlation coefficient or delay based on its vertical and horizontal coordinates. Same below. (**A**) indicates the rEMG–AC in group E; (**B**) indicates the rEMG–AC in group W; (**C**) indicates the ΔtEMG–AC in group E; (**D**) indicates the ΔtEMG–AC in group W. The abscissa in the figure represents the position and axis of the accelerometer (refer to the annotation in Figure 2); the ordinate represents the eight muscles of the forearm. The time delay values are calculated based on the “EMG–AC”; a negative value indicates that the EMG was earlier than AC_acc_, while a positive value indicates a lag.

As shown in Figure 5A, the values of rEMG–AC were above 0.85 in group E, which means the correlations were excellent. Among them, the rEMG–AC values of BR and FCR with the AC_acc_ of each axis were slightly lower, below 0.9. The rEMG–AC values between BR and the AC_acc_ of each axis were even lower, with cross-correlation coefficients all below 0.87. For ΔtEMG–AC, only E–x and E–z showed large values, and the values of ΔtEMG–AC were zero on both the ulnar and radial sides of the wrist (Figure 5C). In group W, the values of rEMG–AC were above 0.84, indicating excellent cross-correlations. Similar to group E, the rEMG–AC between BR and the AC_acc_ of each axis was also relatively low, as was FCR. In addition, in group W, the rEMG–AC between PT and the AC_acc_ of each axis also showed relatively low degrees of cross-correlation (r < 0.88), which differed from group E (Figure 5B). The ΔtEMG–AC values in group W were almost zero, and only a 0.6 ms time delay was seen between the AC_acc_ of E–x and the EMG activity of FCR (Figure 5D).

#### 3.3.2. The Cross-Correlation Coefficients of EMG and Acceleration Change (rEMG–AC)

**Figure 6:**
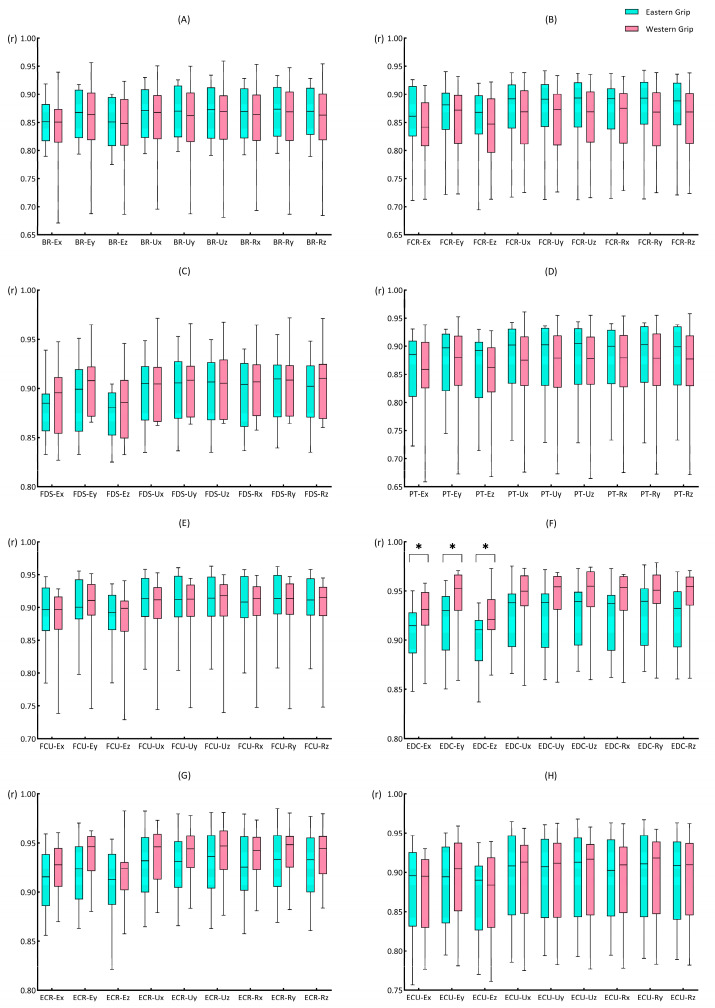
The rEMG–AC of forehand stroke with Eastern and Western grips. (**A**–**H**) represent the cross-correlation coefficients between the EMG of BR, FCR, FDS, PT, FCU, EDC, ECR, ECU and the nine sets of acceleration data, respectively. The data are presented as box plots, and the * indicates a significant difference (*p* < 0.05).

The Mann–Whitney U test results showed that in the comparison between groups E and W, the data of rEDC–Ex (*p* = 0.035), rEDC–Ey (*p* = 0.016), and rEDC–Ez (*p* = 0.039) were significantly different. The rEDC–Ex (0.91, 0.041; 0.93, 0.034 [median and interquartile range for group E; median and interquartile range for group W; applies throughout]), rEDC–Ey (0.93, 0.055; 0.95, 0.036), rEDC–Ez (0.91, 0.042; 0.92, 0.031) of group W were all higher than those of group E (Figure 6F).

#### 3.3.3. The Time Delays of EMG and Acceleration Change (ΔtEMG–AC)

For the ΔtEMG–AC indicator, no significant difference was found (Supplementary Figure S3) between groups E and W. The activity of the muscles preceded the AC_acc_, and the time delays were slight (<20 ms), with most of the data showing a result of 0 ms.

### 3.4. The Cross-Correlation Coefficients and Time Delays of EMG and Jerk

#### 3.4.1. General Description

**Figure 7:**
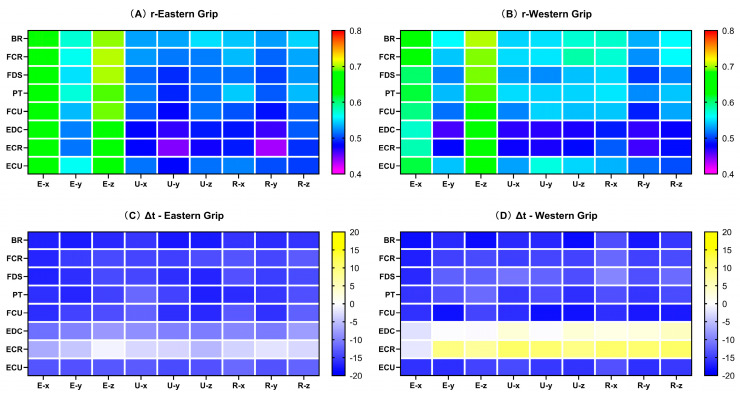
The cross-correlation coefficients and delays of EMG–jerk. (**A**) indicates the rEMG–jerk in group E; (**B**) indicates the rEMG–jerk in group W; (**C**) indicates the ΔtEMG–jerk in group E; (**D**) indicates the ΔtEMG–jerk in group W. The abscissa in the figure represents the position and axis of the accelerometer (refer to the annotation in Figure 2); the ordinate represents the eight muscles of the forearm. The time delay values are calculated based on the “EMG–jerk”. A negative value indicates that the EMG was earlier than Jerk, while a positive value indicates a lag.

The result shows that the rEMG–jerk values in group E (Figure 7A) ranged from 0.4 to 0.7, with moderate to strong cross-correlations. The rEMG–jerk of the elbow X-axis and Z-axis with EMG signals were strong. Among them, the rEMG–jerk between the elbow X-axis and EMG of the eight muscles were above 0.63, and the values between the elbow Z-axis and EMG of the eight muscles showed the highest results (>0.66). For time delay, the EMG activity of the eight forearm muscles all preceded the Jerk change. Among them, the ΔtEMG–jerk of the EMG of ECR and EDC with jerk values of each axis were the smallest, within 12 ms (Figure 7C). In group W, the rEMG–jerk values ranged from 0.46 to 0.70, with moderate to strong cross-correlations. Similar to group E, in group W, the jerk value and EMG also showed strong cross-correlations in the X-axis and Z-axis of the elbow joint, and the values of the elbow joint Z-axis were the highest. Overall, the rEMG–jerk values of group W were higher than those of group E (Figure 7B). Group W and E had similar results for the time delay, but the data related to ECR and EDC are different. The results of group W showed that the EMG activity of ECR and EDC lagged behind the Jerk change (Figure 7D).

#### 3.4.2. The Cross-Correlation Coefficients of EMG and Jerk (rEMG–jerk)

**Figure 8:**
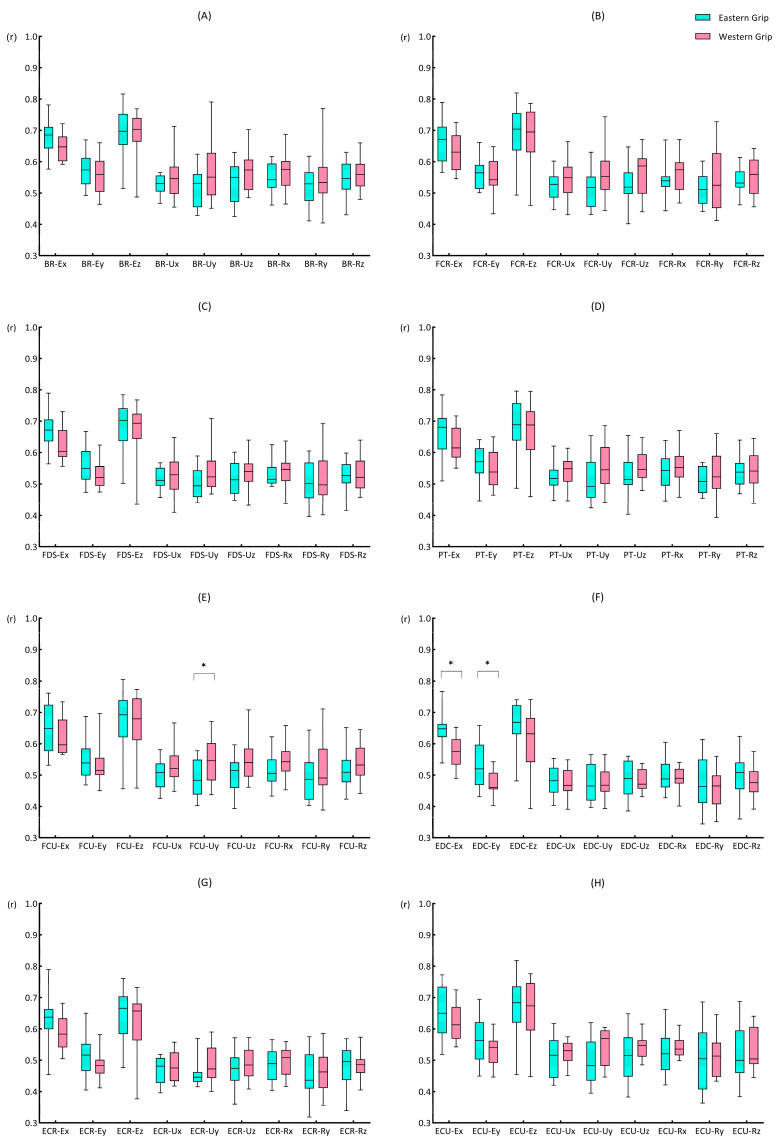
The rEMG–jerk during forehand stroke with Eastern and Western grips. (**A**–**H**) represent the cross-correlation coefficients between the EMG of BR, FCR, FDS, PT, FCU, EDC, ECR, ECU and the nine sets of acceleration data, respectively. The data are presented as box plots, and the * indicates a significant difference (*p* < 0.05).

Mann–Whitney U test results showed that (Figure 8) rFCU–Uy (*p* = 0.044), rEDC–Ex (*p* = 0.001), and rEDC–Ey (*p* = 0.021) showed significant differences between groups E and W. The rFCU–Uy of group W (0.55, 0.119) was greater than that of group E (0.48, 0.111). The rEDC–Ex (0.65, 0.042) and rEDC–Ey (0.52, 0.129) of group E were both higher than the rEDC–Ex (0.58, 0.081) and rEDC–Ey (0.46, 0.053) of group W.

#### 3.4.3. The Time Delays of EMG and Jerk (ΔtEMG–jerk)

**Figure 9:**
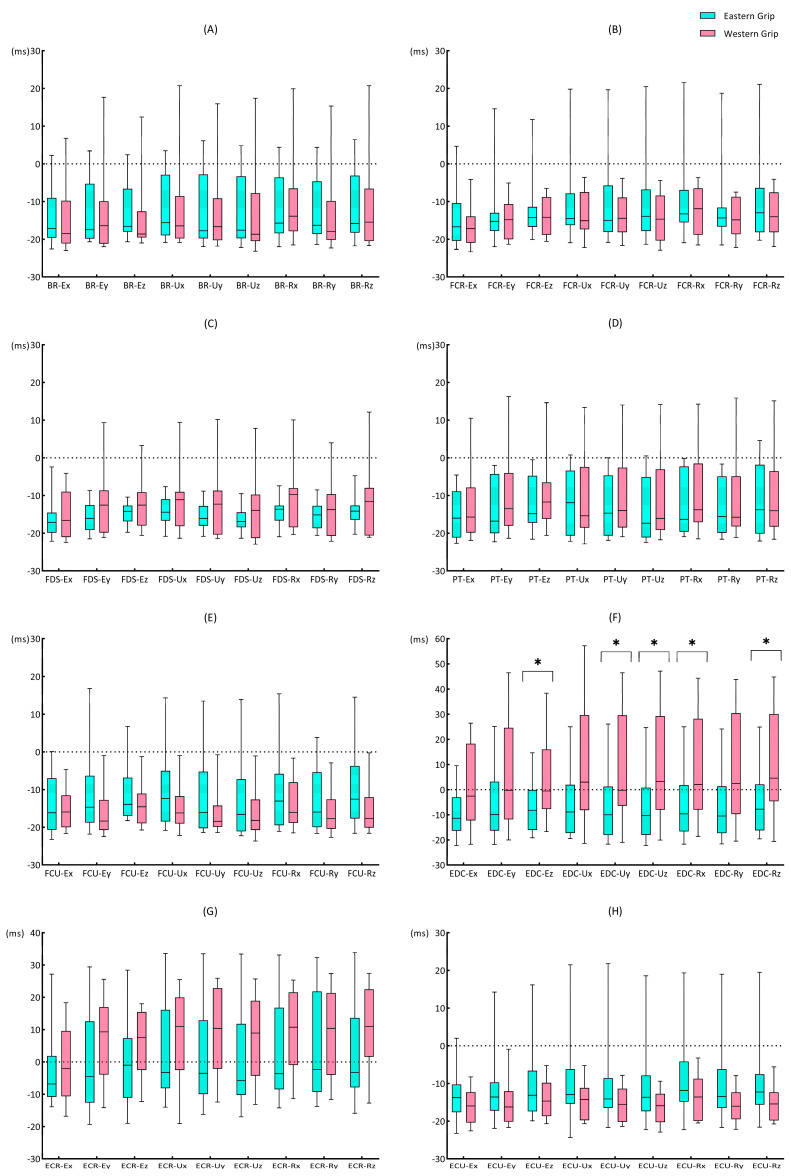
The ΔtEMG–jerk during forehand stroke with Eastern grip and Western grip. (**A**–**H**) represent the time delays between the EMG of BR, FCR, FDS, PT, FCU, EDC, ECR, ECU and the nine sets of acceleration data, respectively. The data are presented as box plots, and the * indicates a significant difference (*p* < 0.05). The time delay values are calculated based on the “EMG–jerk”; a negative value indicates that EMG was earlier than Jerk, while a positive value indicates a lag.

For the ΔtEMG–jerk, there were significant differences (Figure 9) between groups E and W in five groups of results: EDC–Ez (*p* = 0.044), EDC–Uy (*p* = 0.031), EDC–Uz (*p* = 0.039), EDC–Rx (*p* = 0.039), and EDC–Rz (*p* = 0.048). Of all the results with significant differences [EDC–Ez (−8.21, 15.98; −0.54, 23.77), EDC–Uy (−10.00, 19.29; −0.25, 35.96), EDC–Uz (−10.33, 18.83; 3.29, 37.27), EDC–Rx (−9.63, 18.48; 2.04, 36.14), EDC–Rz (−7.79, 18.33; 4.58, 34.75)], group E showed greater time delays than group W, and muscle activation preceded the Jerk change. Although the time delay values of group W were small, EDC–Uz, EDC–Rx, and EDC–Rz showed a characteristic of muscle activity behind the Jerk change.

### 3.5. The Cross-Correlation Coefficients and Time Delays of EMG and EMG

#### 3.5.1. General Description

**Figure 10:**
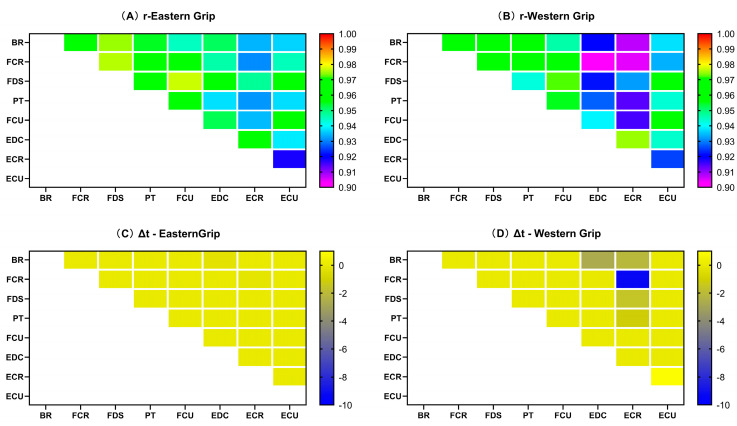
Coefficient of cross-correlation and delay of EMG–EMG. (**A**) indicates the rEMG–EMG in group E; (**B**) indicates the rEMG–EMG in group W; (**C**) indicates the Δt of EMG–EMG in group E; (**D**) indicates the Δt of EMG–EMG in group W. The time delay values are calculated based on the “abscissa–ordinate”; a negative value indicates that the EMG of abscissa was earlier than that of ordinate, while a positive value indicates a lag.

In general, the EMG signals of all muscles (Figure 10A, B) in groups E and W exhibited excellent levels of cross-correlation (rEMG–EMG > 0.9), and the ΔtEMG–EMG (Figure 10C, D) between all muscles were small (<10 ms), indicating a high degree of synchronization of forearm muscles activity. In group E, the rEMG–EMG values between the ECR and the other muscles (except EDC) were slightly lower (Figure 10A), but the time delays were smaller (Figure 10C). In group W, the cross-correlations between EDC and ECR with other muscles were slightly lower (Figure 10B), while ECR and other muscles had larger time delays (Figure 10D).

#### 3.5.2. The Cross-Correlation Coefficients of EMG and EMG (rEMG–EMG)

**Figure 11:**
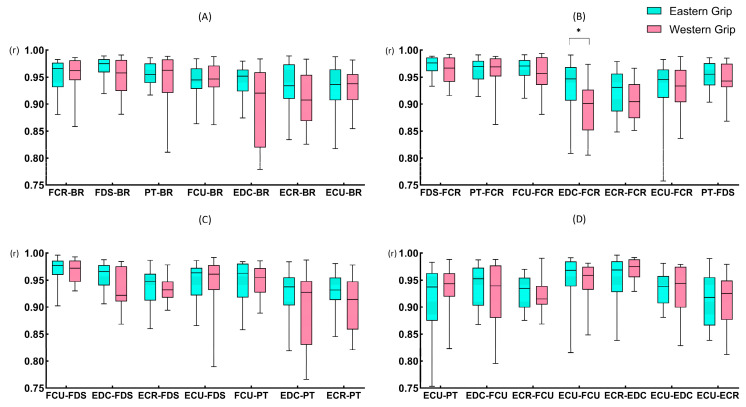
The rEMG–EMG during forehand stroke with Eastern grip and Western grip. (**A**–**D**) represent the cross-correlation coefficients of the EMG of two different target muscles, respectively. The data are presented as box plots, and the * indicates a significant difference (*p* < 0.05).

The Mann–Whitney U test results (Figure 11) show a significant difference in rEDC–FCR (*p* = 0.039) in the comparison between groups E and W. The rEDC–FCR of group E (0.95, 0.06) was greater than that of group W (0.90, 0.075).

#### 3.5.3. The Time Delays of EMG and EMG (ΔtEMG–EMG)

For the time delay indicators, no significant difference was found in the comparison between groups E and W (Supplementary Figure S4).

## 4. Discussion

This study selected the Eastern and Western grips for analysis, as they are the two most representative forehand stroke grips in tennis. We mainly explored the muscle activities, the distribution and attenuation of impact loads, and the contribution of muscles to the impact load attenuation after hitting with different grips. The results showed some interesting differences between the Eastern and Western grip.

### 4.1. Characteristics and Differences in Muscle Activity

In comparing different grips, the activation degree of FCU showed significant differences, with the RMS in group E significantly higher than in group W. This was because the FCU not only flexes the wrist but also participates in the adduction of the wrist. Similarly, the Eastern grip has a characteristic of wrist flexion and adduction and a more fixed position on the ulnar side. Therefore, a greater degree of FCU activation was displayed, which is consistent with the findings of Forman et al. [44]. Their study assessed the activity of the forearm muscles during dynamic radial–ulnar deviation of the wrist joint with different forearm postures (pronation/supination) in 12 healthy male college students. It was found that there was a significant difference in muscle activity of the FCU during ulnar deviation and radial deviation, and the EMG activity during ulnar deviation was higher than that during radial deviation. Furthermore, the study also found that the EMG activity level of the FCU was unrelated to different forearm postures. It suggests that the difference in FCU muscle activity we observed between the Eastern and Western grips may be due to wrist deviation rather than forearm rotation. In addition, group E showed higher activation of the pronator and wrist flexor muscles than group W, while in group W, the wrist extensor muscles showed higher activation. This condition is consistent with our understanding of the characteristics of Eastern and Western grips. That is, the forearm is in a pronation position with flexion of the wrist joint in the Eastern grip, and the Western grip form shows the supination of the forearm and extension of the wrist.

The indicators of cross-correlation coefficient and time delay of EMG can represent the level of synchronized muscle contraction during movement [39]. According to our results, whether using the Eastern or Western grip, the eight muscles all showed good correlations and short time delays, indicating a high level of synchronized contraction of the forearm muscles. Furthermore, the wrist balance is essential to ensure the success of the grip task [35]. In group E, the cross-correlation coefficient of EDC–FCR was significantly higher than in group W. This is because in the Eastern grip, besides the inherent flexion of the wrist joint, the tendons of the finger flexor muscles also create an additional flexion moment [45] when passing through the wrist joint. Coupled with the large impact force of the ball hitting the racket, co-activation of the agonistic and antagonistic muscles was required to maintain the stability of the wrist joint. Therefore, intensive actions of wrist extensor muscles are necessary [46], which is consistent with the view of Rossi et al. [47]. Their musculoskeletal modeling of the hand also showed that, during tennis forehand stroke, the greater force of wrist extensor muscles (the ECR, ECU, and EDC, etc.) was required to counterbalance the undesirable wrist flexion moment created by finger flexor muscles. Similarly, in several other groups of wrist extensors and flexors (FCR–ECR, FCR–ECU, FCU–ECR, FCU–ECU, EDC–FCU), the cross-correlation values of group E were higher than that of group W, although without statistically significant difference. Therefore, we believe that in the Eastern grip, the activation of forearm extensor muscles mainly counterbalanced the flexion moment at the wrist joint, thereby maintaining wrist stability. Consequently, the wrist flexor and extensor muscles were more synchronously activated. However, in the Western grip, in addition to counteracting the flexion moment, the primary function of wrist extensor muscles was to ensure movement performance. Therefore, although the extensor muscles in the Western grip have higher EMG activity levels than those in the Eastern grip, the synchronization between the extensor and flexor muscles was lower in the Western grip, which can also explain our RMS results. Moreover, the co-activation of the EDC and FCR may also contribute to balancing the moment of ulnar deviation in the Eastern grip and maintaining the stability of the radial side of the wrist. Our research shows that coaches and athletes need to focus on improving the overall strength of forearm muscles, especially achieving the balance of wrist flexors and extensor muscle strength. This balance is important for maintaining wrist stability, thereby ensuring stroke effectiveness and preventing injuries.

### 4.2. Characteristics and Differences in the Distribution and Attenuation of Impact Load

The strength of a shock wave can be assessed by acceleration value [37,48]. Therefore, peak acceleration can indicate the maximum impact load on the human body during movement. In this study, we found that, no matter which grip, the impact load showed significant attenuation from wrist to elbow, similar to the results of backhand strokes by Hennig et al. [17]. However, compared to the Western grip, the ulnar side of the wrist joint suffered greater impact loads when using the Eastern grip, which is one of the factors leading to ulnar side injuries. Tagliafico et al. [1] investigated the association between different grips and wrist injuries in 370 tennis players. Their results showed that the Eastern grip is more likely to cause injury to the radial side of the wrist joint than to the ulnar side, which seems to contradict our results. However, we also found that the amplitude of impact load attenuation on the ulnar side during the Eastern grip was greater than during the Western grip. This means the forearm seems to significantly attenuate impact vibration waves through several active (muscle contraction [4]) or passive (soft tissue [49]) shock-absorbing mechanisms. Meanwhile, our results of the PEAK–jerk also demonstrate that impact load attenuation on the ulnar side is quicker when using the Eastern grip. Therefore, although the ulnar side suffers a greater impact load during the Eastern grip than the Western grip, the impact load attenuates more greatly and rapidly, so the ulnar side did not accumulate excessive load during the eastern grip. Similarly, the western grip also showed similar results on the radial side despite no significant difference in the related impact load indicators. We still observed a trend in which the radial side suffered greater impact loads, but the amplitude and rate of attenuation of impact load were higher than those of the Eastern grip. It indicates that the different injury patterns on wrist joints with different grips may not only be related to the maximum impact load during exercise but also to the amplitude and attenuation rate of impact load.

### 4.3. Relationships and Differences in Muscle Activity and Impact Load Attenuation

Previous studies have shown that hand-held racket vibration is more rapidly damped (about 10 times faster) than freely suspended racket vibration [26,50]. It shows special features in the attenuation mechanism of impact loads when holding the racket. A study by Hoshino et al. [49], using autopsies, proved that soft tissues could absorb impact loads well. Rogowski et al. [18] also pointed out that vibration attenuation seems to be associated with the viscoelastic properties of soft tissues, but it mainly affects the low-frequency parts of the vibration signal. Additionally, the relationship between muscle activity and the attenuation of impact load was also very tight. Skeletal muscles can dissipate the mechanical energy of external impacts by absorbing mechanical shocks [51]. Our results showed a high degree of synchronism between muscle activity and the amplitude of impact load attenuation, and muscle activity also contributes to the rate of impact load change (Jerk).

Regarding the relationship between the muscle activity and the amplitude of impact load attenuation (EMG–AC), we found that the cross-correlation coefficients between EMG–AC were relatively high, all above 0.84, and the time delays were close to 0. These results indicated a high degree of synchronization between muscle activity and the attenuation of impact load. Among them, the cross-correlation coefficients between the EMG of EDC and the amplitude of impact load attenuation in the X, Y, and Z axes of the elbow showed significant differences between the Eastern grip and Western grip (group W > group E). Interestingly, the cross-correlation results of the EMG of EDC and the Jerk of elbow X and Y axes also showed significant differences between the two grips. However, unlike EMG–AC, the results of EMG–jerk only showed a moderate degree of cross-correlation, and the value of the Eastern grip was higher than that of the Western grip. It suggested that the contraction of the EDC may be more focused on the amplitude of impact load attenuation and has less effect on the rate of impact load attenuation. Furthermore, the effects of the EMG activity of EDC on the impact load attenuation in the two grips are also different. In the Western grip, the contraction of EDC was more focused on increasing the amplitude of impact load attenuation, while in the Eastern grip, it was more closely related to the rate of impact attenuation. Previous studies have shown that the extensor carpi radialis brevis (ECRB) is the main contributor to wrist extension [52]. At the same time, EDC and ECU play synergistic roles in wrist extension, while extensor carpi radialis longus (ECRL) contributes more to abduction during wrist extension. Meanwhile, Kelley et al. [53] showed that the activation of ECRB and ECRL exhibited significant differences during stroke in athletes with and without tennis elbow, while no significant differences in EDC activity. It suggests that ECR activity may be related to the Western grip performance and tennis elbow, but EDC may have a different role. Combined with the results of our study, we speculate that the activation of EDC contributed more to attenuating impact loads at the elbow joint. Furthermore, the increased contribution of the EDC to wrist control may reduce the load on ECRB, thereby reducing the risk of pain induction and/or further injury [54].

In the time delay results of the EMG–jerk, there were significant differences between the EMG of EDC and the jerk values of elbow Z-axis, wrist ulnar Y-axis and Z-axis, and wrist radial X-axis and Z-axis. In the Eastern group, the EMG activity preceded the change of Jerk, while in the Western group, the EMG activity lagged behind the Jerk. Although the time delays of EDC activity and other axial Jerks did not show significant statistical differences, they showed similar patterns. It means that the EMG activity of the EDC may be more “pre-emptive” in regulating the attenuation rate of the impact load in the Eastern grip, whereas in the Western grip, it tends to be more “delayed”. The ECR was generally considered to be the main extensor of the wrist joint [52]. Because the characteristics of the Western grip involve wrist extension and abduction, the ECR needs to generate higher activity to complete the movement, and additional activation of EDC may be required to assist wrist extension and reduce the stress of ECR. However, the Eastern grip did not require significant wrist extension, so the EDC was probably more relaxed, which was more conducive to actively performing pre-contraction to smooth the impact load attenuation. A comparative study of the wrist extensor muscles in patients with tennis elbow and healthy people [55] showed that although the coordination patterns of forearm muscles were similar between the two groups, the time delay from EDC contraction to the wrist extension beginning was greater in patients with tennis elbow. Other research has also shown that the mechanical overuse of the wrist extensor tendons is a risk factor for tennis elbow [47]. Therefore, combined with the results of our study, we believe that the Western grip may contain more risk factors for tennis elbow than the Eastern grip. Additionally, Tagliafico [1] also reported that the Western grip style was more dangerous to the wrist, and wrist strength and perfect stroke timing were the keys to avoiding injury.

### 4.4. The Smoothness of Impact Load Attenuation (Jerk)

Our PEAK–jerk and rEMG–jerk results showed that the X-axis of the elbow, the Y-axis of the elbow, and the Y-axis of the wrist ulnar side differed significantly between grips (Figure 6 and Figure 11). The Jerk not only indicates the decay rate of impact load but also serves as a critical indicator for assessing the smoothness of movement [38]. In our study, Jerk can also indicate the smoothness of impact load attenuation. The Eastern grip exhibited lower PEAK–jerk in the elbow X-axis and Y-axis, which indicated that the impact loads attenuated more smoothly at the elbow joint in the Eastern grip. Meanwhile, in the Eastern grip, the cross-correlation coefficients between the EMG of EDC and the Jerk of the elbow X-axis and Y-axis were significantly higher than that in the Western grip. Therefore, we believe that, in Eastern grip forehand strokes, the EMG activity of EDC contributed more to the smoothness of impact attenuation at the elbow joint. In the Y-axis of the wrist ulnar side, the PEAK–jerk of the Eastern grip was greater than that of the Western grip, and the cross-correlation coefficient of FCU–Uy in the Western grip was greater than that of the Eastern grip. It indicated that, in the Western grip, the impact on the Y-axis of the wrist ulnar side attenuated more smoothly, and the FCU contributed more to the smoothness of impact attenuation here. High impact attenuation smoothness means that the bones and joints are protected from the additional impact loads (like the sudden acceleration or braking of a vehicle [56]), which improves sports comfort and reduces the risk of sports injuries. It also provides a novel perspective on the active anti-vibration mechanism of the body.

In summary, our results show the importance of EDC and FCU for impact load attenuation. In particular, EDC significantly affects the amplitude and rate of impact load attenuation in both Western and Eastern grips. Therefore, athletes and coaches should pay special attention to FCU and EDC during training.

### 4.5. Limitations

Our study still has several limitations. Firstly, subject recruitment was difficult due to the limited number of elite tennis players, which resulted in a small sample size for this study, even though it had sufficient statistical power. Secondly, we only used the wireless EMG system and the triaxial accelerometers. Kinematic indicators were not involved, which cannot describe the specific movement characteristics of strokes with different grips. Additionally, most of the forearm muscles are small and densely distributed, so the signals obtained by the wireless EMG system may be affected by cross-talk from the EMG signals of adjacent muscles [57]. Intramuscular electrodes are unsuitable for testing muscle activity during dynamic actions, so a high-density surface EMG system may be a better choice. Lastly, because it has the characteristics of both Eastern grip and Western grip and the injury situation is similar to that of Western grip, our study did not separately investigate the semi-Western grip. However, in modern tennis, more and more amateurs or athletes are accustomed to using the semi-Western grip. In the future, equipment such as motion capture system, high-density surface EMG system, and triaxial accelerometer can be combined to conduct more in-depth research on tennis skills. At the same time, more subjects can be recruited, and more grips and motions can be added to explore the characteristics of tennis in depth.

## 5. Conclusions

This study shows that the FCU exhibited higher levels of EMG activity in the Eastern grip. Moreover, this grip responded to greater impact loads with more substantial and rapid attenuation on the wrist ulnar side. In addition, EDC appeared to contribute more to the amplitude of impact load attenuation in the Western grip and to have a more significant influence on the rate of impact load attenuation in the Eastern grip, especially at the elbow. However, FCU contributed more to the smoothness of impact attenuation during Western grip. Furthermore, in the Eastern grip, the EMG activity of the EDC regulates the attenuation rate of the impact loads earlier, whereas in the Western grip, the EMG activity tends to be more delayed. Our findings suggest that the recruitment of forearm muscles and the impact load attenuation strategies differ between different grips, and EDC plays an unexpectedly important role. It suggests that, besides increasing the general strength of forearm muscles, tennis players and coaches should pay more attention to achieving the balance of wrist flexors and extensor muscle strength and improving the strength of the EDC and FCU. Focusing on these points is significant for attenuating impact loads (both Eastern and Western grips), which can improve sports performance and comfort and prevent sports injuries.

## Data Availability

The data presented in this study are available on request from the corresponding author. The data are not publicly available due to privacy and ethical restrictions, as they contain information that could compromise the privacy of research participants.

## Supplementary Materials

The following supporting information can be downloaded at: https://www.mdpi.com/article/10.3390/life14111433/s1, Figure S1: Schematic diagram of the Eastern and Western grips in tennis; Figure S2: Schematic diagram of the experimental site; Figure S3: The ΔtEMG–AC during forehand stroke with Eastern and Western grips; Figure S4: The ΔtEMG–EMG during forehand stroke with Eastern and Western grips; Table S1: Summary of indicators with significant differences between different grips.
