# Supplementary material for: Characteristics, Relationships, and Differences in Muscle Activity and Impact Load Attenuation During Tennis Forehand Stroke with Different Grips
Source: Life (Basel). 2024 Nov 6;14(11):1433. doi: 10.3390/life14111433 (PMC11595894; doi:10.3390/life14111433)
Supplement: Supplementary file 1 [file life-14-01433-s001.zip › life-3280338-supplementary.pdf]

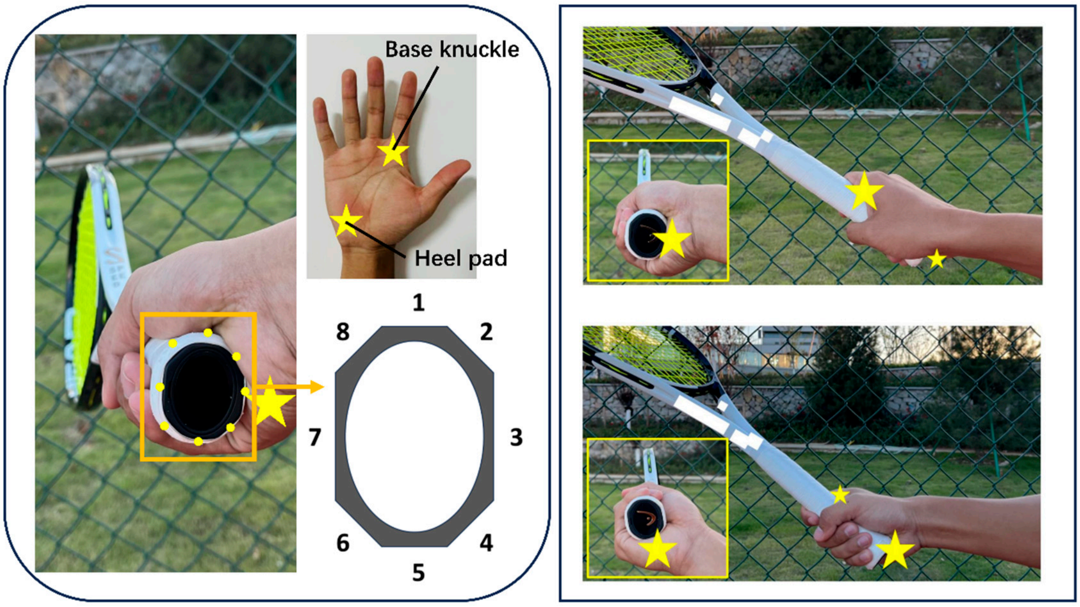

Supplementary Figure S1. Schematic diagram of the Eastern and Western grips in tennis. The left picture showed the eight surfaces of the racket handle and the reference points on the hand (base knuckle of the index finger and heel pad) that were used to identify the different grips. The right picture showed the Eastern (top right) and Western (bottom right) grip. Note that the racket was at the same angle while the player's hand has changed. The yellow pentagrams represented key points of the hand (base knuckle and heel pad).

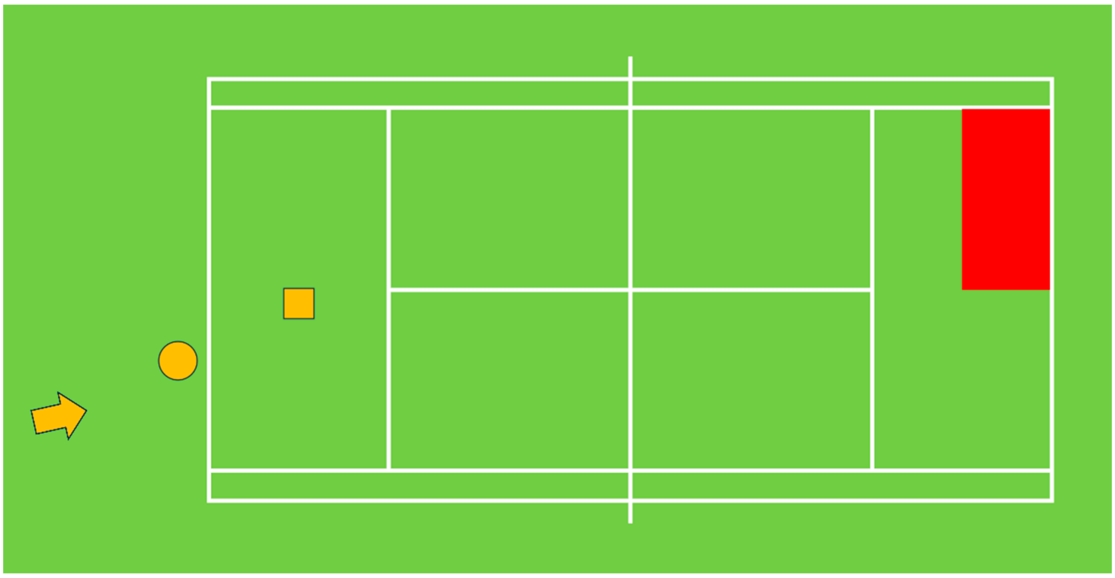

Supplementary Figure S2. Schematic diagram of the experimental site. The red square is the target area defined by the researcher; The yellow square indicates the pitching machine; The yellow circle indicates the position where the subject stands; and the yellow arrow indicates the radar speed detector and direction.

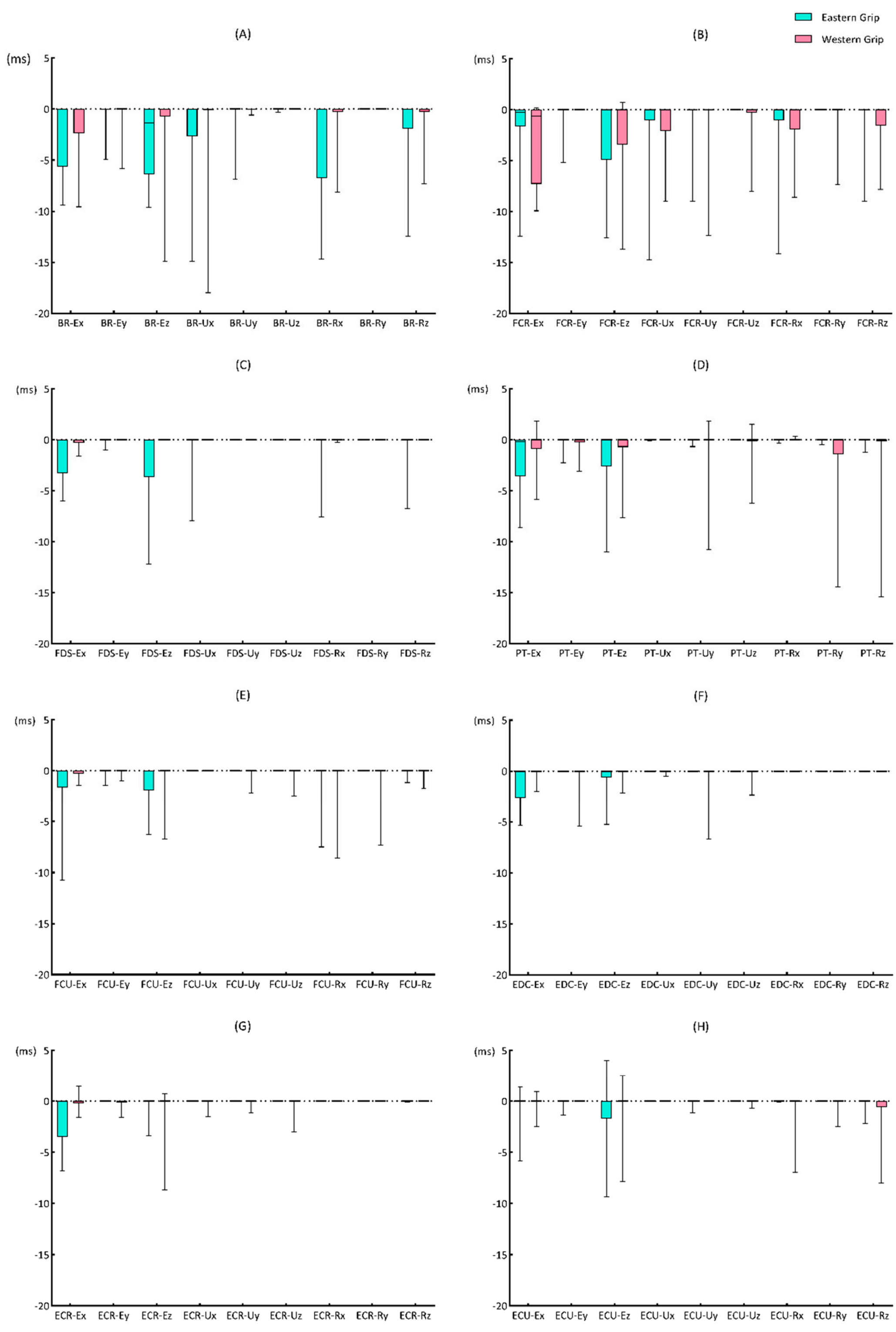

Supplementary Figure S3. The ΔtEMG-AC during forehand stroke with Eastern and Western grips. (A), (B), (C), (D), (E), (F), (G), (H) represent the time delays between the EMG of BR, FCR, FDS, PT, FCU, EDC, ECR, ECU and the nine sets of acceleration data, respectively. The data are presented as box plots, and the * indicates a significant difference ($p<0.05$). The time delay value is calculated based on the "EMG-AC"; a negative value indicates that the EMG was earlier than ACacc, while a positive value indicates a lag.

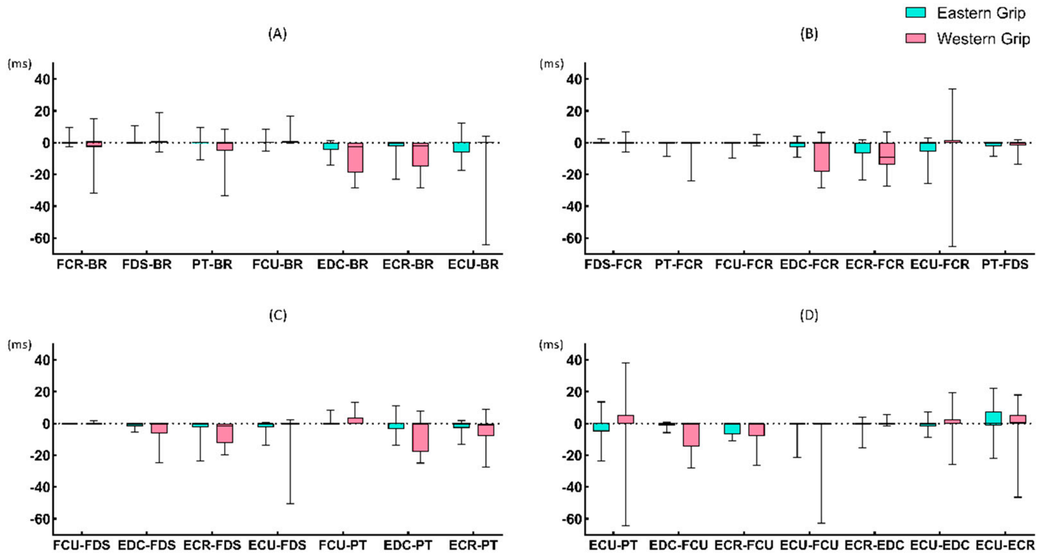

Supplementary Figure S4. The ΔtEMG-EMG during forehand stroke with Eastern and Western grips. The data was presented as box plots. The time delay values are calculated based on the "EMG1-EMG2"; a negative value indicates that EMG1 was earlier than EMG2, while a positive value indicates a lag.

Supplementary Table S1. Summary of indicators with significant differences between different grips.

Classification	Indicator [1]	Eastern grip group	Western grip group	P value
RMS	FCU	0.96±0.30 [2]	0.97±0.21	0.037
PEAK-acc	U-y	27.46,11.99 [3]	15.95,12.93	0.016
PAC-acc	U-y	52.18,25.55	30.09,22.96	0.008
	R-y	36.03,18.69	53.58,23.20	0.048
PEAK-jerk	E-x	0.057,0.024	0.090,0.062	0.048
	E-y	0.202,0.205	0.346,0.247	0.024
	U-y	0.929,0.599	0.485,0.683	0.021
rEMG-AC	rEDC-Ex	0.91,0.041	0.93,0.034	0.035
	rEDC-Ey	0.93,0.055	0.95,0.036	0.016
	rEDC-Ez	0.91,0.042	0.92,0.031	0.039
rEMG- jerk	rFCU-Uy	0.48, 0.111	0.55, 0.119	0.044
	rEDC-Ex	0.65, 0.042	0.58, 0.081	0.001
	rEDC-Ey	0.52, 0.129	0.46, 0.053	0.021
ΔtEMG-jerk	EDC-Ez	-8.21,15.98	-0.54,23.77	0.044
	EDC-Uy	-10.00,19.29	-0.25,35.96	0.031
	EDC-Uz	-10.33,18.83	3.29,37.27	0.039
	EDC-Rx	-9.63,18.48	2.04,36.14	0.039
	EDC-Rz	-7.79,18.33	4.58,34.75	0.048
rEMG-EMG	rEDC-FCR	0.95, 0.06	0.90, 0.075	0.039

[1] The meanings of indicator abbreviations can be found in Figure 1-4,6,8,9,11; [2] Results were calculated by the independent-sample T-test and expressed as mean ± standard deviation; [3] Results were calculated by the Mann-Whitney U test and expressed as the median, the interquartile range.
